# Experiences, Knowledge Gaps and Information Needs of Women in Australia with Transfusion Dependent Thalassaemia in Regard to Fertility and Pregnancy

**DOI:** 10.1007/s10995-023-03683-8

**Published:** 2023-06-05

**Authors:** Hannah K Matthiesson, Vasili Berdoukas, Esther M Briganti

**Affiliations:** 1 Department of Epidemiology and Preventive Medicine, School of Public Health, Monash University, 553 St Kilda Road, Melbourne, VIC 3004 Australia; 2 Department of Pediatrics, University of Southern California, 1975 Zonal Avenue, Los Angeles, CA 90033 USA

**Keywords:** Transfusion dependent β-thalassaemia, Fertility, Pregnancy, Contraception, Reproductive healthcare

## Abstract

**Objectives:**

Transfusion dependent β-thalassaemia can have significant effects on fertility and is also associated with significant risks in pregnancy. However, little is known about the perspectives of women living with the condition with regards to reproductive issues. The aim of this study was to assess the experience, knowledge and information needs of Australian women living with transfusion dependent β-thalassaemia in relation to fertility and pregnancy.

**Methods:**

A cross sectional study using an online anonymous survey, self-administered through REDCap, addressing key issues related to the experience, knowledge and information needs of women with transfusion dependent β-thalassaemia. Descriptive and inferential analysis was conducted using STATA.

**Results:**

Sixty participants were included in the analysis. Two-thirds of sexually active, pre-menopausal women were using contraception. Just under half of the participants who were sexually active had children and half had required some form of assisted reproductive technology to achieve a pregnancy. Less than half identified the importance of contraception as part of ensuring optimised pre-pregnancy care, and less than half had accessed pre-pregnancy care. Although there was good understanding of the increased risk of infertility and pregnancy complications, the specific risks and causes of these risks were poorly understood. Around half of the participants indicated they wanted more information on these medical issues.

**Conclusions for practice:**

Our study demonstrated significant concerns and knowledge gaps in Australian women with transfusion dependent β-thalassaemia with regards to disease-specific issues related to fertility and pregnancy, and a desire for related patient information.

**Supplementary Information:**

The online version contains supplementary material available at 10.1007/s10995-023-03683-8.

## Significance

*What is already known on this subject?* Transfusion dependent β-thalassaemia has a number of serious complications known to have significant impacts on fertility and pregnancy. Perspectives and knowledge levels of women with transfusion dependent thalassaemia with regards to reproductive issues have been relatively unexplored.

*What does this study add?* There are significant knowledge gaps amongst women with transfusion dependent β-thalassaemia in regards to disease specific reproductive health information, which could impact negatively on reproductive health outcomes, and that concerns and information needs are currently not being adequately addressed.

**Supplementary Information:**

The online version contains supplementary material available at 10.1007/s10995-023-03683-8.

## Introduction

Transfusion dependent β-thalassaemia (TDT) results in a life-long, transfusion-dependent anaemia and consequent iron overload necessitating appropriate iron chelation to prevent a wide variety of complications, many of which have significant impact on fertility and pregnancy outcomes. Hypogonadotropic hypogonadism due to pituitary iron overload is a common complication and therefore many women, particularly in the setting of significant iron overload during childhood and adolescence, have delayed puberty and sexual maturation, and primary or secondary amenorrhea (Castaldi et al., 2016; Carlberg et al., 2018). Consequently, medical intervention is often required to conceive (Kyriakou et al., 2009; Srisukh et al., 2016; De Sanctis et al., 1988). Additionally, pregnancies associated with TDT are considered ‘high-risk’ as they can be associated with a number of complications, most significantly cardiac failure, diabetes mellitus and thrombotic events for the mother, and intrauterine growth restriction, preterm birth, and vertical transmission of blood-borne viruses for the fetus (Origa et al., 2010; Bajoria et al., 2009; Tuck et al., 2005; Eleftheriou et al., 1998; Petrakos et al., 2016). Many of these risks can be mitigated with appropriate management of the disease prior to conceiving and during the pregnancy, in particular reducing iron overload with adequate chelation (Origa et al., 2019; Saxena et al., 2017).

Women with TDT have been found in previous studies to be as likely to be in a relationship, be sexually active and desire children as women without TDT (Psihogios et al., 2002). Studies have indicated that greater focus is needed in understanding condition-specific reproductive healthcare needs, and in the provision of patient information for women with chronic diseases so that informed decisions about fertility matters, including contraception, can be facilitated (Holton et al., 2018). Therefore, the aim of this study was to investigate the experiences, knowledge gaps and concerns in regard to fertility and pregnancy of women in Australia with TDT, in order to better understand their reproductive healthcare needs and develop disease specific patient and healthcare resources to improve fertility and pregnancy outcomes.

## Methods

### Study Design

An online self-administered anonymous survey was developed based on a similar survey tool (Charron-Prochownik et al., 2006) and using information derived from a literature review of specific clinical issues related to fertility and pregnancy in TDT. The survey could not be accessed without the provision of informed consent. It consisted of 84 questions across six domains: demographic characteristics, TDT related clinical characteristic, contraception and pregnancy experiences, contraception knowledge, fertility and pregnancy knowledge, and information preferences for patient related education about reproductive health. The survey consisted of quantitative (fixed choice responses and Likert scales) and qualitative (free-text response) questions. Study data were collected and managed using REDCap electronic data capture tools hosted at Monash University (Harris et al., 2009). Ethics approval was granted by Monash University Human Research Ethics Committee in September 2019 (Project ID: 19,768). Details of the survey are provided in Online Resource 1.

All adult women living in Australia with a formal diagnosis of TDT were considered eligible for the study. The survey was advertised in tertiary hospitals with thalassaemia treatment centres and via thalassaemia support group websites and social media pages. No official registry of people with TDT exists in Australia, but it is estimated that there are approximately 150 adult women with TDT. Therefore, based on a confidence level of 95% and margin of error of 10%, the calculated survey sample size was 59.

### Data Analysis

Number of cases and percentages for categorical data and median and range for non-parametric continuous data were used to describe the data. Univariate logistic regression was used to examine the relationship between demographic and clinical characteristics with the clinical outcomes of interest, with results presented as odds ratios (OR) with 95% confidence intervals (CI). The level of significance for all analyses was 0.05. Data analysis was conducted using Stata version 16 (Stata Corp, College Station, TX, USA).

## Results

### Sample and Response

A total of 87 individuals accessed the survey link online, of which 76 consented to take part in the study and proceeded to the survey. Fifty-one surveys were completed in full, and another 25 were only partially completed. Of these 25, in 16 surveys only the demographic section was completed and these were excluded from the study, and in the remaining 9 surveys the majority of survey was completed (> 80%) and they were included in the study. Therefore, responses from a total of 60 surveys were analysed. Table 1 summarises participant demographic and clinical characteristics.

**Table 1:** Demographic and clinical characteristics of participants who completed the survey

Age	number (%)
18–30 years	19 (31.7%)
31–39 years	9 (15.0%)
40–49 years	27 (45.0%)
50 + years	5 (8.3%)
Education (n = 50)	
Secondary school	9 (18.0%)
Trade qualification, certificate or diploma	17 (34.0%)
University degree or higher	24 (48.0%)
Frequency of transfusions	
Every 2 weeks	4 (6.7%)
Every 3 to 4 weeks	51 (85.0%)
Less frequently than monthly	5 (8.3%)
Chelation	
Only deferoxamine	4 (6.7%)
Only deferiprone	1 (1.7%)
Only deferasirox	36 (60.0%)
Deferoxamine & deferiprone	5 (8.3%)
Deferoxamine & deferasirox	5 (8.3%)
Deferiprone & deferasirox	6 (10.0%)
Deferoxamine & deferiprone & deferasirox	3 (5.0%)
Disease severity (self-reported)	
Iron overload status (n = 53)	
Ferritin, ug/l^a^	900 (200, 5700)
Ferritin < 1,000 ug/l^b^	33 (62.3%)
Self-reported complications	
Delayed puberty n = 54	18 (30.0%)
Hypogonadism n = 48	20 (33.3%)
Infertility n = 43	22 (36.7%)
Diabetes mellitus n = 57	9 (15.0%)
Heart disease n = 56	5 (8.3%)
Liver disease n = 57	7 (11.7%)
Osteoporosis n = 55	32 (53.3%)
Fractures n = 57	12 (20.0%)
Hypothyroidism n = 53	13 (21.7%)
Hypoparathyroidism n = 48	10 (16.7%)
Presence of any complication	41 (68.3%)
Number of complications^a^	2 (0, 8)

n = 60 unless otherwise state

variables presented as number (%) unless otherwise stated

^a^Median (range)

^b^As per Australian guidelines (Ho et al., 2011)

### Reproductive Health Experiences

Of the pre-menopausal (52/60), sexually active women (45/52) who were not trying to conceive (36/45), 47.2% (17/36) were using contraception. The reported contraceptive methods used were condoms (6/17; 35.3%), the combined oral contraceptive pill (5/17; 29.4%), the progesterone-only pill (3/17; 17.6%), tubal ligation (1/17; 5.9% ) and vasectomy (1/17; 5.9%). Reported reasons for not using contraception were infertility (9/19; 47.4%), contraception was not medically recommended (3/19; 15.8%), inability to tolerate contraception (1/19; 5.9%) and religious beliefs (1/19; 5.9%). Information about contraception had been sought by 49.1% (28/57) of the women, the main source being general practitioners, gynaecologists, and haematologists. The information obtained was reported as consistent in 75.0% (21/28) and useful in 71.4% (20/28).

Among the women who had achieved a pregnancy (25/60; 41.7%), there was a total of 54 pregnancies resulting in 45 children (3 women had twin pregnancies) and 12 spontaneous miscarriages. The number of pregnancies ranged from 1 to 4, with a median of 3 pregnancies. Fertility treatment was required in 76.0% (19/25) of the women for 40.7% (22/54) of the pregnancies. Fertility treatments undertaken were ovulation induction (13/22, 59.1%), in-vitro fertilisation (6/22, 27.3%), intracytoplasmic sperm injection (2/22, 9.1%) and intrauterine insemination (1/22, 4.5%).

Pre-pregnancy care had been accessed by 44.4% (24/54) of women, with haematologists, thalassaemia nurses and fertility specialists being the most common reported sources of information. The information obtained was reported as consistent in 87.5% (21/24) and useful in 75.0% (18/24). The main reported reasons for not accessing pre-pregnancy care were lack of knowledge of its availability (7/30; 23.3%), lack of knowledge as to its requirement (3/30; 10.0%), lack of pregnancy planning (2/30; 6.7%), planned pregnancy that happened sooner than expected (1/30; 3.3%) and not wanting a pregnancy different from women without thalassaemia (1/30; 3.3%). For the women who had been pregnant, 52% (13/25) experienced maternal complications and 28% (7/25) experienced fetal complications during their pregnancies. Parity ranged from one to three, with a median of 2 children.

For woman who had not been pregnant (35/60, 58.3%), the main reported reasons were that they did not want children (13/35; 37.1%), they had not yet tried to get pregnant (10/35; 28.6%), they had tried without fertility treatment but were unable to achieve a pregnancy (2/35; 5.7%) and they had tried with fertility treatment but were unable to achieve a pregnancy (4/35; 11.4%).

### Contraception, Fertility and Pregnancy Knowledge

Knowledge as to the safety of contraception use in women with TDT was generally low except for the COCP. While 76.8% (43/56) considered the COCP to be safe, 50.0% (28/56), 46.4% (26/56) and 42.9% (24/56) considered the progesterone only pill, contraceptive implants and intrauterine devices safe, respectively. The importance of contraception for planned pregnancy was noted by 50.0% (28/56) of women, with only 7.1% (4/56) thinking contraception was unnecessary due to TDT being associated with infertility. The increased risk of infertility associated with TDT was known by 66.7% (38/57) of women. However, only 40.4% (23/57) were aware that a normal serum ferritin could significantly increase the chance of achieving normal fertility. Pre-pregnancy care was thought to be important for a healthy pregnancy and healthy off-spring by 75.9% (41/54) and 72.2% (39/54), respectively. The potential for significant health problems in pregnancy for mothers was correctly identified by 37.0% (20/54) of women, and for the off-spring by 31.5% (17/54). The importance of achieving a normal ferritin level was noted by 67.7% (36/54) for a healthy pregnancy and by 53.7% (29/54) for having healthy off-spring.

The proportion of women who correctly answered 25 questions assessing fertility and pregnancy knowledge in TDT is shown in Table 2. The median score for the total correct answers was 15 (range 4 to 25). Significant predictors of poor fertility and pregnancy knowledge (median score < 15) were younger age (OR 3.6 [95% CI: 1.1, 11.7], P = 0.035 for 18–30 years of age compared to older than 30 years of age) and lower levels of attained education (OR 8.5 [95% CI: 1.5, 46.7], P = 0.014) for secondary school compared to post-secondary school education). A trend for poor fertility and pregnancy knowledge was seen in relation to employment status (OR 3.37 [95% CI: 0.81, 14.1] P = 0.096 for not in full-time compared to full-time employment), presence of thalassaemia complications (OR 3.1 [95% CI: 0.8, 11.2, P = 0.088] for presence of any complication compared to no complications) and gravidity (OR 3.1 [95% CI: 0.8, 11.2, P = 0.080] for history of no pregnancy compared to any pregnancies).

**Table 2:** Fertility and pregnancy knowledge of women with TDT (n = 55)

	Correct response
Use of contraception in women with thalassaemia is limited due to safety	50.0% False
Testing for thalassaemia and other blood disorders in partners is not recommended	92.6% False
Genetic counselling is recommended prior to planning for pregnancy	83.3% True
If you had an unplanned pregnancy this could cause health problems for you?	37.0% True
Control of ferritin levels is important for normal fertility	61.1% True
Control of ferritin levels is important for a healthy pregnancy	66.7% True
Control of ferritin levels is important for a healthy baby	53.7% True
Inadequate control of thalassaemia before falling pregnant increases the risk of health problems for the baby	67% True
Inadequate control of thalassaemia before falling pregnant increases the risk of health problems for the mother	87% True
Change in chelation medications is not required during pregnancy	78% False
Women with thalassaemia should not take pregnancy multi-vitamins	45% False
Women with thalassaemia should take the same amount of folate as other women during pregnancy	20% False
Thalassaemia does not increase the risk of birth defects	27% False
Thalassaemia related complications that women have before falling pregnant can affect the health of the baby	76% True
Thalassaemia related complications that women have before falling pregnant can affect the health of the mother	80% True
Low blood calcium levels in women with thalassaemia can cause negative effects on the pregnancy	31% True
An underactive thyroid in women with thalassaemia can cause negative effects on the pregnancy	31% True
Women with thalassaemia have an increased risk of developing diabetes during pregnancy	42% True
Inadequate control of thalassaemia during pregnancy increases the risk of health problems for the baby	78% True
Inadequate control of thalassaemia during pregnancy increases the risk of health problems for the mother	87% True
Thalassaemia related complications during pregnancy can affect the health of the baby	85% True
Thalassaemia related complications during pregnancy can affect the health of the mother	87% True
Women with thalassaemia have an increased risk of having a small baby	20% True
The greatest risk for women with thalassaemia when pregnant relates to heart disease	25% True
Women with thalassaemia can breastfeed	29% True

### Information Preferences

Significant concerns were noted in regards to reproductive health issues, with 55.0% (33/60) of women stating they worried ‘a moderate amount’ to ‘a lot’ that they could develop health problems during a pregnancy, and 65.0% (39/60) having concerns about the development of health problems in their off-spring.

Table 3 outlines the information needs of women with TDT. For women of reproductive age, 66.7% (32/48) thought there was benefit in receiving information about planning and preparing for pregnancy, but only 39.6% (19/48) want to receive such information. For women wanting to receive information, 84.2% (16/19) wanted to receive it at least annually, with the preferred source of information being their haematologist (6/19, 31.6%), or through a professional thalassaemia support group (5/19; 26.3%). The most popular requested format was web-based written information (6/19, 31.6%), email (5/19, 26.3%) or face-to-face (5/19, 26.3%).

**Table 3:** Information needs of women with TDT (n = 60)

Type of information	number (%) of participants
Thalassaemia and contraception	27 (45.0%)
Thalassaemia and fertility	35 (58.3%)
Thalassaemia and infertility	32 (53.3%)
How to manage thalassaemia to prepare for pregnancy	38 (63.3%)
How to manage thalassaemia during pregnancy	40 (66.7%)
Risks of complications for the baby in a woman with thalassaemia	35 (58.3%)
Medication changes during pregnancy	33 (55.0%)
Thalassaemia and breastfeeding	31 (51.7%)
How to manage thalassaemia after childbirth	30 (50.0%)
Importance of testing for thalassaemia and other blood disorders in partners of women with thalassaemia	23 (38.3%)
Importance of counselling prior to pregnancy with your partner	22 (36.7%)

Additional comments provided by some participants (13/60, 21.7%) are provided in Online Resource 2 - Thematic Summary of Additional Responses. The three main themes identified related to a lack of information, or desire for more information, related to TDT specific reproductive health, the importance of receiving information at an early age, and previous negative experiences.

## Discussion

The major findings of this study were that while many women with TDT have had, or plan to have children, there were significant knowledge gaps and concerns about the effect of TDT on fertility and pregnancy, and a discordance between disease knowledge and health seeking behaviour.

While there was moderate understanding that TDT can contribute to infertility, there was poor knowledge of the mechanisms leading to this, in particular the negative effect of iron overload. This finding was consistent with another Australian study which reported that only a third of its participants with clinically significant haemoglobinopathy were aware iron overload could result in infertility (Crowther et al., 2013). While the majority of our participants understood the importance of pre-pregnancy care in thalassaemia, only half of the women who had previously been pregnant reported accessing pre-pregnancy care prior to conceiving. A similar finding has been described in a study of women with type 1 or type 2 diabetes, where although 80% of participants understood diabetic related pregnancy risks, less than half had sought preconception advice prior to their pregnancies (Hibbert et al., 2018).

Lower uptake of contraception use was reported in our study compared to the general Australian population (Richters et al., 2003) and compared to Australian women with other chronic diseases (Hibbert et al., 2018). Several studies have shown that women with chronic diseases commonly underestimate their fertility potential or are unsure of which contraceptives are safe to use, contributing to a lower uptake of contraception use (Hibbert et al., 2018; Johannesson et al., 1998; Hull et al., 2000). While less than 10% of women in this study reported a presumption of reduced fertility, close to half of participants were unsure of the safety of the wide range of contraception available in Australia in TDT. The COCP was the most popular reported contraceptive method used in this study, with very low uptake of long-acting contraceptives, such as contraceptive implants and intrauterine devices. This finding suggests the need for both further patient education about the importance of contraceptive use to reduce the risk of an unplanned pregnancy, and education of patients and healthcare professionals about the safety and effectiveness of various contraceptives in TDT, given the significant risks of an unplanned pregnancy in TDT.

A concerning finding was the lack of knowledge about the risks of unplanned pregnancies in TDT. Given the known higher rates of unplanned pregnancies in women with chronic diseases (Holton et al., 2018) and the high-risk nature of unplanned pregnancies for women with TDT, this knowledge gap is important to address. Although there were good levels of understanding of the importance of partner testing, our cohort actually had poorer knowledge compared with an Australian study conducted in 1998 in which all but two of 106 participants understood the importance of partner testing (Psihogios et al., 2002). This finding may reflect the change in demographics of people with TDT in Australia in the last 20 years with increased proportion of people with TDT from non-English speaking backgrounds, or possibly an assumption by treating healthcare professionals that the importance of genetic counselling and partner testing is already well known. While overall knowledge about the effects of TDT on pregnancy was limited, it did vary significantly across specific topics and subgroups of women. For example, there was good understanding that TDT is associated with maternal and fetal complications in general, but there was poor knowledge of the TDT specific complications women were at greater risk of, and the role that iron overload plays in these complications. This phenomenon has been shown amongst women with chronic diseases, with previous studies reporting a lack of disease specific knowledge on pregnancy outcomes (Holton et al., 2018; Chuang et al., 2010). Socioeconomic status and educational attainment are well known to be key social determinants of health outcomes (Marmot et al., 2006; Hardman et al., 2020), a finding also seen in this study such that women with lower levels of employment and educational attainment were found to have lower levels of TDT knowledge. Concerningly, for women in whom good knowledge of the negative impacts of TDT was most relevant - younger women, women who had not previously been pregnant and women with TDT complications - lower levels of TDT knowledge was demonstrated.

One important area of poor knowledge was with regards to the safety of breastfeeding. Breast feeding rates in women with TDT have been found to be lower than the general population and may be a result of poor knowledge regarding its safety (Origa et al., 2010). While current guidelines encourage breastfeeding for women with TDT, a recent Italian study reported both patient and medical staff misconceptions relating to concerns of transmission of viral hepatitis contributing to low breast-feeding rates (Origa et al., 2010). A strong desire for further information about a wide range of topics was reported in the study, including information about infertility and management of TDT before, during and after pregnancy, but less so for management relating to partner screening and pregnancy counselling. Recommendations were made by participants that information should be made available at a younger age.

This study has several important strengths, in particular its multi-institutional representation, the inclusion of about a third of all women in Australia with TDT, and being one of the few studies to specifically explore women’s perspective of issues related to fertility and pregnancy in TDT. Although the online nature of the study allowed for nationwide dissemination of the survey, a limitation was the inability to ensure responses were from unique individuals. A further limitation was possible selection bias, with a notable representation of women who had previously had children, and under-representation of women from more marginalised populations, including individuals from culturally or linguistically diverse backgrounds, from rural and regional areas, and with a lower educational level. These biases would most likely result in an observed over-estimation of knowledge level and potentially more favourable reported perspectives in respect to issues of fertility and pregnancy. Furthermore, the sample size was too small to adequately explore the independent association of risk factors with knowledge gaps and concerns identified.

In conclusion, our study revealed that there is a need for improved knowledge amongst women with TDT in regards to TDT specific reproductive health information, and that concerns and information needs are not currently being adequately addressed. Patient information should focus on the major knowledge gaps which relate to the (i) role of iron overload as the primary cause of both infertility and the major complications in pregnancy (cardiac failure and diabetes) associated with poor obstetric outcomes (ii) TDT specific clinical management needs before, during and after pregnancy, in particular the importance of optimally reducing iron overload with chelation therapy from an early age to prevent infertility, and prior to pregnancy to reduce the risk of cardiac failure and diabetes (iii) importance and safety of contraception to allow for appropriate pre-pregnancy planning and treatment to address iron overload as well as other factors impacting on pregnancy outcome, and (iv) importance and safety of breast-feeding. Barriers to access to necessary medical care need further exploration given the discordance between disease and treatment knowledge and health seeking behaviour to optimise health.

### Electronic Supplementary Material

Below is the link to the electronic supplementary material.

Supplementary Material 1

## Data Availability

On application.
