# Supplementary material for: Experiences, Knowledge Gaps and Information Needs of Women in Australia with Transfusion Dependent Thalassaemia in Regard to Fertility and Pregnancy
Source: Matern Child Health J. 2023 Jun 5;27(11):1961–7. doi: 10.1007/s10995-023-03683-8 (PMC10564654; doi:10.1007/s10995-023-03683-8)
Supplement: Supplementary file 1 — Supplementary Material 1 [file 10995_2023_3683_MOESM1_ESM.docx]

# Experiences, knowledge gaps and information needs of women in Australia with transfusion dependent thalassaemia in regard to fertility and pregnancy. Maternal and Child Health Journal

Hannah K Matthiesson^1^, Vasili Berdoukas^2^, Esther M Briganti^1^

1. Department of Epidemiology and Preventive Medicine, School of Public Health, Monash University, 553 St Kilda Road, Melbourne VIC 3004, Australia.
2. Department of Pediatrics, University of Southern California, 1975 Zonal Avenue, Los Angeles, California 90033, United States.
Corresponding author: Esther M Briganti: [esther.briganti@monash.edu](mailto:esther.briganti@monash.edu)

Online Resource 1 – Study Survey
Online Resource 2 - Thematic Summary of Additional Responses (n=13)

##

## Online Resource 1 – Study Survey *Domain 1: Demographics*

1. How old are you?

- 18-24 years
- 25-30 years
- 31-34 years
- 35-39 years
- 40-44 years
- 45-49 years
- 50 or more years

2. In which state or territory do you live?

- ACT
- SA
- NSW
- Tasmania
- NT
- Victoria
- Queensland
- WA

3. What is the geographic location of where you live?

- Metropolitan
- Regional
- Remote
- Not sure

4. What is your country of birth?

5. What is your mother’s country of birth?

6. What is your father’s country of birth?

7. What is your level of education?

- Year 10, 11 or equivalent
- Year 12 or equivalent
- Certificate, diploma or trade qualification
- University degree or higher
- Other

8. What is your current employment status?

- Student
- Full time home duties
- Working full time
- Unemployed
- Working part time or casual
- Retired
- Parental leave from work
- Other

*Domain 2: Thalassaemia Related Question**s*

1. What type of beta thalassaemia do you have?

- Major
- Intermedia
- Not sure

2. How frequently do you usually have blood transfusions?

- Every 2 weeks
- Every 3 to 4 weeks
- Every 3 to 6 months
- Every 6 to 12 months
- Less than once a yea
- Never had a blood transfusion before
- Other

3. What is your current iron chelation treatment?

- Only deferoxamine
- Only deferiprone
- Only deferasirox
- Combination deferoxamine AND deferiprone
- Combination deferiprone AND deferasirox
- Combination deferoxamine AND deferasirox
- Combination deferoxamine AND deferiprone AND deferasirox
- Other
- Unsure

4. What is your ferritin level within the last 6 months?

5. Which thalassaemia complications are you known to have? Option = Yes/No/Do not know

- Heart disease
- Inadequate development at puberty
- Liver disease
- Low sex hormone levels
- Diabetes
- Infertility
- Underactive thyroid
- Osteoporosis
- Underactive parathyroid (low calcium level)
- Fractures

6. In the last 12 months, how often have you seen these health care professionals for thalassaemia management. Please state the number of visits. Options = 0/1-3/4-6/7-10/11-12/12+

- Haematologist
- GP
- Thalassaemia nurse
- Cardiologist
- Endocrinologist
- Liver specialist

*Domain 3: Fertility And Pregnancy Questions*

1. Are you currently using contraception?

- Yes
- No

2. If no, what is your primary reason for this

- Currently trying to conceive
- Not required (post-menopausal)
- Not medically recommended
- Unable to tolerate
- Not required (infertile)
- Not sexually active
- Religious beliefs
- Other

3. What type of contraception are you using?

- Combined oral contraceptive pill
- Tubal ligation
- Progesterone-only pill
- IUD
- Partner uses condoms
- Contraceptive implants
- Partner has had vasectomy
- Hormonal vaginal ring
- Other

4. Are you currently trying to conceive/achieve a pregnancy?

- Yes
- No

5. Have you required fertility treatment currently or in the past to achieve a pregnancy?

- Yes
- No

IF YES,

5a. Was it successful?

- Yes
- No

5b. What type of fertility treatment have you needed (can choose more than one)?

- Ovulation induction with tablets or injections
- Intrauterine insemination
- In vitro fertilisation
- In vitro fertilisation with ICSI

5c. Did you require?

- Donor egg
- Donor sperm
- Donor embryo
- None of the above

6. Have you been pregnant before?

- Yes
- No

7. How many times have you been pregnant?

8. Have you ever had a miscarriage

- Yes
- No

9. How many miscarriages have you had?

10. Do you have children?

- Yes
- No

11. How many children have you had?

12. For any of your pregnancies, did you experience any pregnancy complications

- Yes
- No
- Do not know

13. For any of your pregnancies, did the baby experience any complications?

- Yes
- No
- Do not know

14. Have you adopted children?

- Yes
- No

15. Have you used a surrogate to have children?

- Yes
- No

15a. IF you haven’t had children?

- This is because you do not want/have not considered having children
- This is because you and your partner have not yet tried to get pregnant
- This is because you tried without fertility treatment but were unable to achieve a pregnancy
- This is because you tried with fertility treatment but were unable to achieve a pregnancy
- Other

*Domain 4: Knowledge About Contraception*1. How important do you think contraception is for a woman with thalassaemia?

- Not at all
- A little
- Somewhat
- A moderate amount
- A lot

2. Would using contraception prevent an unplanned pregnancy?

- Not at all
- A little
- Somewhat
- A moderate amount
- A lot

3. Have you ever discussed the use of contraception with one of your health care team, friends or family?

- Yes
- No

IF YES
Was this with a…

- Haematologist
- GP
- Thalassaemia nurse
- Cardiologist
- Endocrinologist
- Liver specialist
- GP
- Gynaecologist
- Fertility specialist
- Other person with thalassaemia
- Friend
- Internet/social media
- Family member
- Other

3b Was the information you received consistent?

- Yes
- No
- Unsure

3c. Was the information what you wanted to know?

- Yes
- No
- Unsure

IF NO,

3d. Do you want to discuss the use of contraception?

- Yes
- No

3e. Who would you prefer to discussed the use of contraception with?

- Haematologist
- GP
- Thalassaemia nurse
- Cardiologist
- Endocrinologist
- Liver specialist
- GP
- Gynaecologist
- Fertility specialist
- Other person with thalassaemia
- Friend
- Internet/social media
- Family member
- Other

4. In terms of the use of contraception, as far as you are aware, women with thalassaemia. Options = True/False/Do not know

- Can use the combined oral contraceptive pill
- Can use the IUD
- Can use the progesterone-only pill
- Have limited choices of contraception
- Can use contraceptive implants
- Do not need to use contraception as they are unable to get pregnant
- Can use hormonal vaginal rings

5. As far as you are aware, for women with thalassaemia. Options = True/False/Do not know

- Testing for thalassaemia and other blood disorders in their partners is not recommended.
- Genetic counselling is recommended prior to planning for pregnancy
- Are at increased risk of infertility
- It is possible to have a healthy baby
- Thalassaemia-specific advice from a health care professional before planning a

pregnancy is needed

*Domain 5: Knowledge About Pregnancy And Thalassaemia*

1. Have you heard of pre-pregnancy care? (also known as pre-pregnancy planning, preconception care or preconception planning)

- Yes
- No

2. How important do you think pre-pregnancy care (specialist thalassaemia medical care and advice related to pregnancy) for a woman with thalassaemia?

- Not at all
- A little
- Somewhat
- A moderate amount
- A lot

3. Have you ever discussed pre-pregnancy care with one of your health care team, friends or family?

- Yes
- No

IF YES

3a. Was this with a…

- Haematologist
- GP
- Thalassaemia nurse
- Cardiologist
- Endocrinologist
- Liver specialist
- GP
- Gynaecologist
- Fertility specialist
- Other person with thalassaemia
- Friend
- Internet/social media
- Family member
- Other

3b. Was the information you received consistent?

- Yes
- No

3c Was the information what you wanted to know?

- Yes
- No

IF NO,

3d Do you want to discuss pre-pregnancy care?

- Yes
- No

3e. Who would you prefer to discuss pre-pregnancy care with?

- Haematologist
- GP
- Thalassaemia nurse
- Cardiologist
- Endocrinologist
- Liver specialist
- GP
- Gynaecologist
- Fertility specialist
- Other person with thalassaemia
- Friend
- Internet/social media
- Family member
- Other

4. If you had an unplanned pregnancy, do you think this could cause health problems for you?

- Not at all
- A little
- Somewhat
- A moderate amount
- A lot

5. If you had an unplanned pregnancy, do you think this could cause health problems for your baby?

- Not at all
- A little
- Somewhat
- A moderate amount
- A lot

6. If you have previously tried to get pregnant or have been pregnant, did you receive any advice about pre-pregnancy care?

- Yes
- No

IF YES,

6a. Who provided the advice regarding pre-pregnancy care?

- Haematologist
- GP
- Thalassaemia nurse
- Cardiologist
- Endocrinologist
- Liver specialist
- GP
- Gynaecologist
- Fertility specialist
- Other person with thalassaemia
- Friend
- Internet/social media
- Family member
- Other

IF NO,

6b What were the reasons for not receiving pre-pregnancy care? Options = True/False

- I did not know that I required specific advice before planning for pregnancy
- Negative experiences previously when discussing this topic with health care professionals
- I did not know pre-pregnancy care was available
- Too difficult to get my thalassaemia under control for pregnancy
- My pregnancy was not planned
- I already knew what I needed to do
- My pregnancy was planned, but I fell pregnant sooner than expected
- Didn’t want a pregnancy that was different from women without thalassaemia
- Practical issues such as time, cost or easy access of pre-pregnancy care
- Worried or afraid of what I might learn about the problems of pregnancy and thalassaemia
- No services were available in my area
- Didn’t have access to health care professionals I felt comfortable with
- Other

7. If you have ever been pregnant/tried to conceive before, did you and your partner undergo screening for thalassaemia and other blood disorders?

- Yes
- No

IF NOT,

7a. What were your reasons for this? Select all that apply

- I have not been pregnant/tried to get pregnant before
- I did not know screening was available
- I did not know it was necessary
- Practical issues e.g. time, cost, location
- The pregnancy was not planned
- Other

8. Would you say that getting pre-pregnancy care and preparing for pregnancy would help you have a healthy pregnancy?

- Not at all
- A little
- Somewhat
- A moderate amount
- A lot

9. Would you say that getting pre-pregnancy care and preparing for pregnancy would help you have a healthy baby?

- Not at all
- A little
- Somewhat
- A moderate amount
- A lot

10. Would you say that having a ferritin level in the target improves your chances of normal fertility?

- Not at all
- A little
- Somewhat
- A moderate amount
- A lot

11. Would you say that having a ferritin level in the target range before becoming pregnant improves your chances of having a healthy pregnancy?

- Not at all
- A little
- Somewhat
- A moderate amount
- A lot

12. Would having a ferritin level in the target range before becoming pregnant improves your chances of having a healthy baby?

- Not at all
- A little
- Somewhat
- A moderate amount
- A lot

13. How difficult would it be for you to obtain a ferritin level in target range when planning a pregnancy?

- Not at all
- A little
- Somewhat
- A moderate amount
- A lot

14. How difficult would it be for other women with thalassaemia to obtain a ferritin level in target range when planning a pregnancy?

- Not at all
- A little
- Somewhat
- A moderate amount
- A lot

15. If you were to develop health problems during pregnancy, how serious do you think those health problems could be?

- Not at all
- A little
- Somewhat
- A moderate amount
- A lot

16. If your baby was to develop health problems during pregnancy, how serious do you think those health problems could be?

- Not at all
- A little
- Somewhat
- A moderate amount
- A lot

17. How much do you worry (or have worried in the past) that you could develop health problems during pregnancy?

- Not at all
- A little
- Somewhat
- A moderate amount
- A lot

18. How much do you worry (or have worried in the past) that your baby could develop health problems during your pregnancy?

- Not at all
- A little
- Somewhat
- A moderate amount
- A lot

19. What do you believe your risk of developing health problems during pregnancy is compared to other women of your age who do not have thalassaemia?

- Much lower
- Somewhat lower
- The same
- Somewhat higher
- Much higher

20. What do you believe your baby’s risk of developing health problems during pregnancy is compared to other babies whose mothers do not have thalassaemia?

- Much lower
- Somewhat lower
- The same
- Somewhat higher
- Much higher

21. As far as you are aware, Options = True/False/Do not know

- Women with thalassaemia should take the same amount of folate as other women during pregnancy
- Women with thalassaemia should not take pregnancy multi-vitamins
- Change in chelation medications is not required during pregnancy
- Inadequate control of thalassaemia before falling pregnant increases the risk of problems for the mother
- Inadequate control of thalassaemia before falling pregnant increases the risk of problems for the baby
- Inadequate control of thalassaemia during pregnancy increases the risk of problems for the mother
- Inadequate control of thalassaemia during pregnancy increases the risk of problems for the ba
- Complications that women have before falling pregnant can affect the health of the mother
- Complications that women have before falling pregnant can affect the health of the baby
- Complications that women have during pregnancy can affect the health of the mother
- Complications that women have during pregnancy can affect the health of the baby
- The greatest risk for women with thalassaemia who are pregnant relates to heart disease
- Thalassaemia does not increase the risk of birth defects
- Women with thalassaemia have an increased risk of having a large baby
- Women with thalassaemia have an increased risk of having a small baby
- Women with thalassaemia are at an increased risk of developing diabetes in Pregnancy
- An underactive thyroid in women with thalassaemia can cause negative effects on the pregnancy
- Low blood calcium levels in women with thalassaemia can cause negative effects of the pregnancy
- Women with thalassaemia are recommended to breastfeed

*Domain 6: Obtaining Information About Prenancy For Women With Thalassaemia*

1. Do you see a benefit in receiving regular reminders about planning and preparing for pregnancy?

- Yes
- No

2. Do you want to receive regular reminders about planning and preparing for pregnancy?

- Yes
- No

2a. What is your preferred frequency?

- Every 3 months
- Every 6 months
- Every year
- Every second year
- Every 3-5 years

2c. What is you preferred source?

- Haematologist
- Thalassaemia nurse
- Other specialist
- GP
- Thalassaemia support groups
- The internet/social media
- Other

2d. What is your preferred format?

- One-on-one advice
- Group seminar
- Webinar
- Pamphlet or booklet
- DVD
- Web-based written information
- Email
- Text message
- Smart phone app
- Other

3. What information would you specifically be interested in knowing in terms of pregnancy and thalassaemia? Options = Yes/No

- Thalassaemia and contraception
- Thalassaemia and fertility
- Thalassaemia and infertility
- How to manage thalassaemia to prepare for pregnancy
- How to manage thalassaemia during pregnancy
- Risks or complications related to thalassaemia and pregnancy
- Thalassaemia-related medication changes required during pregnancy
- Thalassaemia and breastfeeding
- How to manage thalassaemia after childbirth
- Importance of testing for thalassaemia and other blood disorders in partners of women with thalassaemia
- Importance of counselling prior to planning for pregnancy with your partner

4. Do you have any extra comments/questions that you would like the investigators to know about any of these topics?

Online Resource 2 - Thematic Summary of Additional Responses (n=13)

*Major Theme 1: Lack of information/desire for more information*

“I don't really know about having babies and thalassemia but [I] worry all the time [about] when I might have a baby. No one has told me about this” aged 18-24 years

“I would like to know more [about how] to have a baby without problems. No one talks to me about this.” aged 18-24 years

“This isn't a topic that is brought up enough in my opinion and there isn't enough information out there.” aged 18-24 years

“I'd love more information on fertility and how other patients [with thalassaemia] experience this.” aged 25-29 years

“I would have liked to have known about developing liver disease and blood clots in pregnancy and the effect that would have had on my pregnancy and my baby.” aged 40-44 years

“Information to potential parents [is] highly important - parents with or without thalassaemia.” aged 44-49 years
“When I went through it there was not enough information or education about pre-pregnancy planning or pregnancy care. I have always thought that there is a big gap in the system for thalassaemia patients when it comes to family planning and care before, during, and after [pregnancy].” aged 44-49 years

*Major Theme 2: Importance of receiving information at an early age*

“It's important for young girls and boys to take their medication so they too can have a fruitful life. I really stress that parents need to be thoroughly educated to do the right thing by their child/children.” aged 35-40 years

“Information needs to be given to all the younger kids with thalassemia. It is a bit late for someone after the problems have occurred.” aged 44-49 years

*Major Theme 3: Previous negative experiences*

“I was told by an endocrinologist at 19 [years old] that I could not have children. By 30 years old after 3 rounds of ovulation induction and 2 pregnancies I had 2 beautiful daughters. I wish I had known what was possible. Those years between 19 and my first pregnancy were some of the hardest because I lacked hope.” aged 30-34 years

“Having a baby when you have thalassemia is very scary and most doctors do not know what to do.” aged 35-40 years

## “Too late for me. We always thought that we could never have a family. Good to see there is someone interested in helping people with thalassaemia have normal lives.” aged 50+ years
